# Comprehensive Approaches to Aspiration Pneumonia and Dysphagia in the Elderly on the Disease Time-Axis

**DOI:** 10.3390/jcm11185323

**Published:** 2022-09-10

**Authors:** Takae Ebihara

**Affiliations:** Department of Geriatric Medicine, Graduate School of Medicine, Kyorin University, Tokyo 181-8611, Japan; takae-ebi@ks.kyorin-u.ac.jp; Tel.: +81-422-47-5511; Fax: +81-422-44-0849

**Keywords:** aspiration pneumonia, pneumonia-related sarcopenia, TRP agonist, nutrition, time-axis

## Abstract

Pneumonia in the elderly has been increasing on an annual basis. To a greater or lesser extent, aspiration is a major contributor to the development of pneumonia in the elderly. Antimicrobials alone are not sufficient for the treatment of pneumonia, and the condition may become intractable or even recur repeatedly. In addition, some patients with pneumonia may have no problems with eating, while others are unable to receive the necessary nutrition due to severe dysphagia. It has recently been found that pneumonia decreases both the muscle mass and strength of the swallowing and respiratory muscles, a condition named pneumonia-associated sarcopenia. This contributes to a pathophysiological time-axis of aspiration pneumonia and dysphagia in the elderly, in which silent aspiration leads to the development of pneumonia, and further to dysphagia, malnutrition, and low immunity. Therefore, it is recommended that the treatment and prevention of developing pneumonia should also differ according to an individual’s placement in the disease time-axis. In particular, approaches for preventing aspiration based on scientific findings are able to be implemented at home.

## 1. Introduction

As Sir Osler stated, “Pneumonia is an old man’s friend”. Pneumonia among the elderly has been increasing in advanced countries, where populations are ageing at an accelerating rate. As most deaths from pneumonia in the elderly are attributed to aspiration pneumonia (AsP), its diagnosis, treatment, and prevention are important clinical topics. Clinically, AsP can be divided into ‘overt aspiration’, in which the aspiration is evident, and ‘silent aspiration’, in which the aspiration is not apparent. Videofluoroscopy is capable of detecting both overt and silent aspiration, but is unable to detect “micro-aspiration”; that is, the aspiration of small amounts of oropharyngeal secretions due to a depressed swallowing reflex during sleep. Micro-aspiration may or may not present with symptoms suggestive of aspiration, such as coughing. In particular, micro-aspiration without symptoms (i.e., silent aspiration) is important in the development of pneumonia. On the other hand, choking during a meal due to aspirated food debris or its detection in the sputum (macro-aspiration) is considered overt aspiration. As elderly patients with pneumonia often present with non-specific symptoms, such as general malaise, impaired consciousness, and loss of appetite, the onset of pneumonia may only be detected after a chest X-ray; moreover, the disease is often severe [1]. Therefore, it is important to determine the presence or absence of silent aspiration and the means for its prevention for the treatment of pneumonia in the elderly.

## 2. Prevalence

Pneumonia is a disease with high mortality and morbidity worldwide. In Japan, pneumonia is the leading cause of death in people aged 65 years and over, and is particularly prominent in men aged 80 years and over [2].

Despite AsP generally being more likely to occur in the elderly, the prevalence of AsP may be under-estimated. The prevalence of AsP in the USA has been estimated to be 5–15% among hospitalized patients with community- and hospital-acquired pneumonia [3,4]. Meanwhile, in a cross-sectional national survey of Japan, the prevalence rates of AsP in hospitalized community- and hospital-acquired pneumonia were 60.1% and 86.7%, respectively; this further increased with age, accounting for about 85% in those aged 80–89 years and more than 90% in those aged 90 years and older [5]. This Japanese survey involved a swallowing function test, in order to detect not only overt aspiration but also silent aspiration, and it was found that aspiration is greatly associated with the cause of pneumonia in the elderly. From this study report, it may be no exaggeration to say that most pneumonia in the elderly is AsP.

## 3. Mechanism

**Figure 1:**
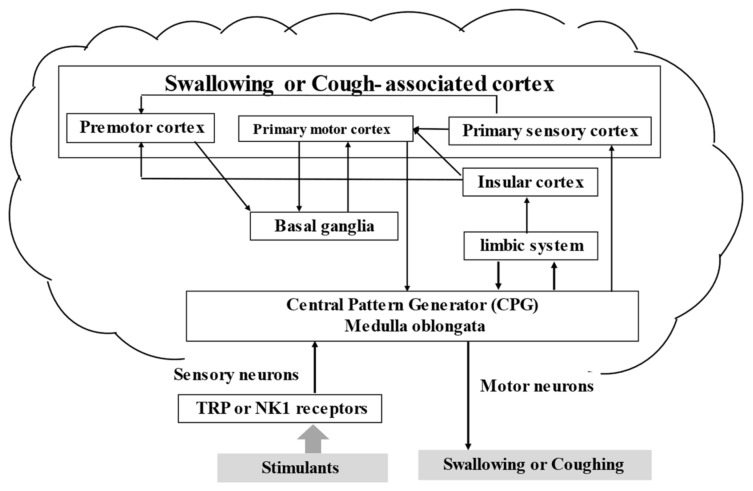
Swallowing, coughing, and the brain. Abbreviations: TRP, transient receptor potential; NK1, neurokinin 1.

The main responsible factor in the development of AsP is the deterioration of the swallowing reflex and cough reflex sensitivity due to reduced release of the neurotransmitter substance P from the nerve endings of the glossopharyngeal and the vagal nerves [6] (Figure 1).

### 3.1. Cough Reflex Sensitivity

Coughs are provoked by the ligand substance P of the neurokinin 1 receptor, located in the respiratory tract and in the nucleus tractus solitarii of the brainstem [7,8,9]. In support, selective neurokinin 1 antagonists have been found to completely suppress coughing in a study in guinea pigs [10,11]. Another animal study has shown that aerosols of a substance P antagonist inhibited acetylcholine- and histamine-induced coughs, which are bronchoconstricting agents [12]. Additionally, coughing was induced through the inhalation of substance P by patients with pulmonary fibrosis [13].

However, contrary to general expectations, geriatric wards—where pneumonia patients are mainly hospitalized—are often quiet, with few coughing patients. The cough reflex sensitivity induced by chemical stimulants is typically blunted in elderly patients with repeated pneumonia [14]. Specifically, the threshold for coughing to citric acid mist in elderly people with repeated pneumonia was a concentration greater than 1.35 log mg mL^−1^, compared to less than 0.5 log mg mL^−1^ in non-pneumonia patients [15].

### 3.2. Swallowing Reflex

Similar to the blunted cough reflex sensitivity, the triggering of the swallowing reflex is also blunted in elderly patients with repeated pneumonia; in particular, the latency of the swallowing reflex in patients with repeated pneumonia has been found to be >5 s [15]. The provocation of the swallowing reflex was mediated by substance P in a capsaicin concentration-dependent manner [16].

### 3.3. Lacunar Infarction and the Upper Respiratory Protective Reflexes

The blunted swallowing reflex and cough reflex sensitivity have been associated with lacunar infarction in the basal ganglia [17]. In patients with bilateral lacunar infarction, the swallowing reflex latency is significantly higher than in other patients with unilateral or no lacunar infarction, and the swallowing reflex latency gradually increases, not only during the day but also at night, with a greater rate of change from daytime than in other patients. Additionally, the incidence of aspiration—assessed using indium chloride—was reported to be higher in elderly patients with bilateral lacunar infarction [18]. The cough reflex sensitivity in the elderly with lacunar infractions has also been shown to be depressed. Furthermore, both the swallowing and coughing reflexes, among others, were greatly impaired in those who developed AsP [17].

The production of substance P is associated with dopamine metabolism. In animal models pre-treated with dopamine 1 receptor antagonists, the provocation of the swallowing reflex was delayed, and the swallowing frequency was increased with exogenously administered substance P, while substance P antagonists decreased the swallowing frequency. In other words, the swallowing reflex and swallowing frequency are substance P-dependent, while substance P is dopamine-dependent [19]. Taken together, the presence of lacunar infarcts may suggest that the blunted swallowing reflex latency and cough reflex sensitivity are due to the reduced release of substance P.

### 3.4. Brain and Swallowing

Reduced activity in the insular cortex has been reported in elderly patients with repeated AsP [20]. Functional cerebral imaging during swallowing in healthy adults has shown that the primary motor and sensory areas are most activated, with additional bilateral activation of the anterior cingulate cortex, insula and basal ganglia capsules, and globus pallidus and substantia nigra [21,22,23]. Therefore, the bilateral inactivation of the insula and basal ganglia may contribute to the development of AsP. 

### 3.5. Breathing and Swallowing

Sensory inputs to the sensory nerves reach the medulla oblongata afferentially and integrate the activity of swallowing-related muscles through central pattern generators (CPGs), such that breathing and swallowing can be appropriately coordinated. Natural swallowing begins with laryngeal closure after inspiration (post-inspiratory activity). In more detail, glutamatergic–cholinergic neurons and excitatory networks that generate neural correlates of post-inspiratory activity and inhibitory neural networks, through gamma-aminobutyric acid, contribute to the regulation of timing involved in inspiration [24]. Furthermore, inputs to the CPG from the cortical swallowing area (including the insular cortex) have also been reported to modulate these coordinated respiratory–swallowing movements. In other words, failure to provoke the upper respiratory protective reflex may interfere with the inspiratory and post-inspiratory coordination, which may contribute to the onset of AsP [25].

### 3.6. Comorbidities Modifying the Development of Pneumonia

Several comorbidities contribute to the development of AsP. Gastrointestinal diseases with organic problems and dysfunction are representative comorbidities of AsP. In addition to age-related gastro-oesophageal motility disorders, post-operative status following gastrectomy leads to a predisposition to aspiration of gastrointestinal contents due to reflux [26]. Furthermore, gastric reflux is more likely to occur in elderly people with hiatal hernia of the oesophagus, which has been estimated to affect one in two women over 80 years of age; furthermore, acidity (especially below pH 4) increases the swallowing reflex latency in a negatively pH-dependent manner [27]. The relationship between gastric acid and the swallowing reflex is considered as a factor affecting the occurrence of AsP in this paper. Elderly people often present with chronic constipation, some of whom may vomit due to impaired bowel peristalsis, leading to chemical pneumonitis [28]. Other factors contributing to the development of AsP have been previously reported, including dementia, physical activity impairment, gender, smoking history, decreased oral intake, and drugs such as neuroleptics, which exacerbate the swallowing reflex by lowering serum substance P, resulting in the development of AsP [29,30].

Anticholinergics are also a risk factor for AsP, as the incidence of AsP has been reported to increase in proportion to the intensity of anticholinergic side-effects such as falls, xerostomia, dry eyes, dizziness, confusion, and constipation [31]. It has also been reported that acid suppressants, such as histamine 2 inhibitors and proton pump inhibitors, tend to increase the pH of gastric juice, thereby altering the gastric flora and even the mesopharyngeal microbiota, facilitating the development of AsP [32,33]. Taken together, drugs with antidopaminergic, bowel peristalsis-reducing, or anticholinergic effects, as well as other drugs that alter the gastric microbiota, should be withdrawn and replaced by drugs with other mechanisms of action.

### 3.7. Pneumonia-Associated Sarcopenia

Initially, sarcopenia is defined as an age-related decrease in skeletal muscle mass and strength [34]. As a muscle atrophy other than ageing, it is already well-known in mouse models that hypoxia (8 h/day, 30 cycles/hour, FiO_2_ nadir = 6%) reduces the contractile properties of the diaphragm, especially as a result of muscle atrophy due to increased autophagy, prompting a compensatory metabolic adaptation that increases fatigue tolerance [35].

Since sarcopenia (assessed by grip strength and lower leg circumference) was reported as a risk factor for the development of community-acquired pneumonia in the elderly [36], a number of reports have revealed the association between pneumonia and sarcopenia.

Acute inflammation and chronic inflammation of the lungs are known to cause muscle atrophy [37]. In a retrospective database study of 739 ventilated patients, a reduction in muscle mass was observed, of which about half also had reduced muscle fiber density [38]. Furthermore, it has recently been shown that chronic aspiration and pneumonia cause a reduction in the cross-sectional area of the muscle fibers, including swallowing and respiratory muscles. In an animal model of lipopolysaccharide-induced aspiration, it has been shown that muscle atrophy of the tongue is induced by autophagy, thinning of the diaphragm by inflammatory cytokine production and the ubiquitin–proteasome pathway, and atrophy of the anterior tibialis muscle—which represents skeletal muscle—by both pathways [39].

In elderly patients hospitalized with AsP, the cross-sectional area of the erector spinae muscle, a respiratory accessory muscle, has been reported to decrease by approximately 80% during the time between admission and pneumonia healing. Further, our previous cross-sectional prospective cohort study has shown that both the inspiratory and expiratory respiratory muscle strength and trunk muscle mass were lower in the elderly with pneumonia compared to those with other respiratory diseases, which can, thus, be considered as risk factors for the development and recurrence of pneumonia [40]. With regard to swallowing, it has also been reported that the swallowing and chewing ability is related to the whole-body muscle mass; that poor swallowing ability, as assessed by the water-swallowing test, is mildly related to the upper arm circumference; and that the tongue muscle mass and tongue pressure are significantly lower in people with dysphagia than those without [41,42,43,44,45].

Finally, chronic aspiration and pneumonia lead to decreases in muscle mass and strength, resulting in reduced swallowing capacity, dysphagia due to a reduced expiratory cough peak flow, respiratory muscle fatigue, and reduced respiratory ventilation efficiency. Pneumonia-related sarcopenia leads to a negative pattern of refractory and recurrent pneumonia, as well as further progression of sarcopenia.

### 3.8. End-of-Life in Aspiration Pneumonia and Dysphagia

To date, no studies have examined impaired upper respiratory protective reflexes (e.g., the swallowing reflex and cough reflex sensitivity) as a risk factor for mortality in the elderly. We have recently reported that under-nutrition and an impaired swallowing reflex and cough reflex sensitivity—but not an impaired oral intake capacity—were predictors of death within 90 days in elderly AsP patients [46]. Furthermore, cholecystitis and cholangitis frequently occur in the terminal stages of the disease, which are thought to be due to the loss of gallbladder contractility caused by reduced lipid intake due to fasting, which increases the viscosity of the bile, causing it to become sludgy and unable to be expelled [47].

### 3.9. Pathophysiological Time-Axis of Aspiration Pneumonia and Dysphagia

**Figure 2:**
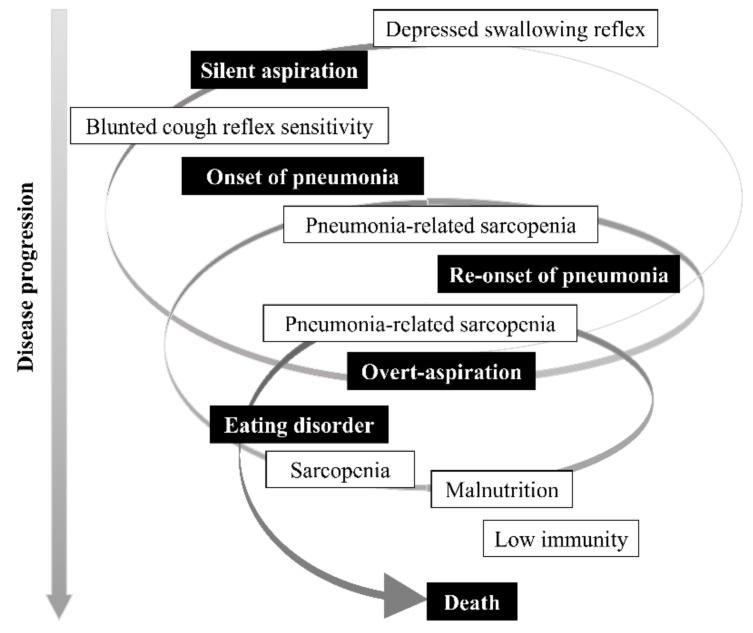
Time-axis of aspiration pneumonia and dysphagia in the elderly.

A noteworthy emerging finding is the time-course of AsP and dysphagia in the elderly: starting with a delayed swallowing reflex, the sensitivity of the cough reflex gradually blunts and AsP develops. The onset of AsP leads to muscle atrophy and weakness of the swallowing and respiratory muscles; that is, a state of pneumonia-associated sarcopenia. Decreases in upper airway protective reflexes and sarcopenia are likely to lead to recurrent pneumonia and further sarcopenia progression, resulting in feeding difficulties and under-nutrition. The inability to meet nutritional requirements, whether by oral intake or other nutritional routes, can be considered terminal (Figure 2).

## 4. Comprehensive Preventive Approach

In addition to adequate antibiotic treatment, comprehensive prophylaxis is important in the treatment of AsP. Specifically, key points include the administration of drugs that promote the production and release of substance P or inhibit its degradation, the improvement of the gastrointestinal motility, the withdrawal of drugs that increase the risk of developing AsP, and nursing and care based on scientific knowledge.

### 4.1. Pharmacological Preventive Approach

#### 4.1.1. Angiotensin-Converting Enzyme Inhibitors

Dry cough is an adverse symptom of angiotensin-converting enzyme inhibitors (ACE-I) [48]. It is well-known that bradykinin and substance P are the causative agents of coughing, and this side-effect is more common in peri- and post-menopausal women [11,48]. In a study of menopausal animal models, ACE-I increased the cough reflex sensitivity, while the coughs were significantly diminished by substance P antagonists [11]. Human studies have reported higher cough reflex sensitivity and substance P concentrations in the sputum in those taking ACE-I compared to non-users [49]. The swallowing reflex in humans was also significantly improved within two weeks after ACE-I medication [50]. In a three-year prospective cohort study of elderly patients with cerebrovascular disease, conducted on the basis of the results of this study, the incidence of pneumonia in the ACE-I treated group was significantly reduced compared with the control, Ca channel blocker, and diuretic groups, suggesting that ACE-I is effective for the prevention of pneumonia [51,52]. Taken together, in clinical situations, ACE-I may be a suitable antihypertensive for hypertensive patients with a history of aspiration or AsP.

#### 4.1.2. Dopamine-Release-Stimulating Agent

As discussed above, dysfunction of the dopaminergic neurons results in a deteriorated swallowing reflex [19], which may lead to the development of pneumonia [17,18]. Amantadine acts as an antagonist at N-methyl-D-aspartate-type glutamate receptors, increasing dopamine release and inhibiting its re-uptake.

In a three-year prospective cohort study, elderly patients with lacunar infarction who were taking amantadine orally had a significantly lower incidence of pneumonia than those not taking the drug, suggesting that dopamine enhancement is effective against the development of pneumonia [53].

#### 4.1.3. Combination of Dopamine-Release-Stimulating Agent and ACE Inhibitor

The treatment of pneumonia with ACE-I or amantadine, as well as antimicrobials, significantly decreased the number of antimicrobial treatment days, hospitalization days, healthcare costs, incidence of methicillin-resistant staphylococcus aureus, and hospital mortality over a one-year period [54]. In another case report, a patient with advanced Alzheimer’s disease was treated with amantadine, levodopa, and ACE-I, and went from being lethargic and anorexic to smiling and talking to their carers, with increased appetite and improved eating disorders, resulting in a recovery of body mass index to the normal range [55]. Therefore, the addition of ACE-I and dopamine-releasing agents may be effective for the prevention of pneumonia in elderly patients with recurrent pneumonia and eating disorders.

#### 4.1.4. Phosphodiesterase-III Inhibitors

It has been reported that a one-year intervention in a cilostazol-treated group (an anti-platelet drug with a phosphodiesterase-III-inhibitory effect) led to an approximately twice as low incidence of pneumonia compared to the non-treated group [56]. Cilostazol not only prevents a high rate of recurrent stroke, but also increases the cerebral blood flow in swallowing-related cerebral regions. It has been reported that cilostazol prevented the incidence of stroke-related pneumonia in patients with acute ischaemic stroke upon tube feeding compared to those without [57,58].

#### 4.1.5. 5-Hydroxytryptamine_4_ Receptor Agonist

A one-year prospective cohort study of post-gastrectomy elderly patients with cerebrovascular disease showed a lower incidence of pneumonia in mosapride-treated patients compared with non-treated patients [59]. Mosapride stimulates 5-hydroxytryptamin_4_ receptors in the gastrointestinal tract’s intrinsic plexus and promotes gastrointestinal motility. In patients receiving gastrostomy feeding, mosapride administration has been shown to be a significant preventive factor for pneumonia [60]. On the other hand, intestinal motility enhancers containing mosapride, unlike cilostazol, did not show a preventive effect on the development of stroke-related pneumonia. In other words, mosapride may prevent the development of pneumonia related specifically to gastrointestinal dysmotility.

### 4.2. Non-Pharmacological Approach: How to Prevent Aspiration at Home

#### 4.2.1. Physical Properties of the Meal

Viscoelasticity, cohesion, and hardness are the three pillars of a texture-modified diet for dysphagia. In clinical settings, foods are typically blended or chopped and served in various thickened liquids (e.g., nectar-, honey-, or paste-thickened), or in the form of a mousse, pudding, or jelly. It has been reported that thickened liquids have significantly faster oral transit times than water but slower pharyngeal transit times, and may leave significant residue [61]. Therefore, can thickening really reduce the incidence of pneumonia? A three-month prospective cohort study has reported that nectar- and honey-thickened liquids decreased aspiration but not the incidence of pneumonia or death, while honey-thickened liquids increased dehydration and anorexia [62]. This means that thickening should not be over-estimated, as texture-modified diets for older people with dysphagia are effective in preventing aspiration, but do not reduce the incidence of pneumonia or death.

#### 4.2.2. Stimulation of Transient Receptor Potential (TRP) Receptor 

The temperature of and spices in meals are also important in preventing aspiration.

##### 4.2.2.1. Temperature of the Meal

The temperature of the meal is an important factor. Temperature-sensitive transient receptor potential (TRP) channels exist in the neural endings of the glossopharyngeal and the vagal sensory branch; TRPV1 receptors respond to hot temperatures (above 60 °C) and TRPM8 receptors to cold temperatures (below 17 °C). Both hot and cold temperatures dramatically sharpen the swallowing reflex, which is delayed by more than 10 s to within the normal range. It is, therefore, advisable to serve food at distinct temperatures, such as hot or cold [63]. For patients with dysphagia, meals served at room temperature are more likely to induce aspiration.

##### 4.2.2.2. Spices

Capsaicin, the pungent component of chilli peppers, is a TRPV1 agonist that triggers the cough or swallow reflex by releasing SP from sensory nerve endings, especially when applied at concentrations of 10^−9^ to 10^−11^ log M/mL, which can improve the swallowing reflex latency in a concentration-dependent manner [16]. Similarly, menthol, a cooling component of mint and a TRPM8 agonist, can also improve the swallowing reflex latency in a concentration-dependent manner at concentrations from 10^−2^ M to 10^−4^ M, especially at concentrations higher than 10^−3^ M, with a significantly shorter reflex than ice water [64].

#### 4.2.3. Aromatherapy

Older people with repeated AsP often show reduced activities of the bilateral insular cortex [20]. Increasing the insular cortical activity, therefore, may serve as a preventive measure against AsP. Piperine, a component of black pepper, is also a TRPV1 agonist that shortens the swallowing reflex, while odour components extracted from the peel activate the insular cortex via the olfactory cortex. In a one-month randomized controlled trial of institutionalized elderly residents, olfactory stimulation with black pepper aroma for one minute before each meal significantly improved the swallowing reflex and the number of swallowing movements, as well as blood flow in the insular cortex upon cerebral single-photon emission computed tomography [65]. The smell-based prevention of aspiration is easy to introduce not only to patients with chronic aspiration or repeated pneumonia, but also to patients with impaired consciousness, patients on ventilators, and patients with severe dysphagia who have difficulty with oral nutrition.

#### 4.2.4. Nutrition

Many people who develop pneumonia are under-nourished compared to those who do not develop pneumonia, and those who repeatedly develop pneumonia have difficulty getting enough calories and nutrients.

Nutritional intensification, calculated as the basal metabolism (Harris–Benedict formula) × physical activity coefficient (e.g., bed-ridden) × stress coefficient (1.1–1.2) using various nutritional routes, was intervened in bed-ridden elderly people for a period of one year [66]. Such an intervention not only reduced the number of pneumonia cases, but also improved other nutritional statuses (serum total protein and albumin levels) without the administration of albumin products. Thus, we re-iterate the need for nutritional treatment in order to help prevent pneumonia in the elderly.

A prospective cohort study considering a nutritional treatment intervention for the elderly at risk of pneumonia presented a significant reduction in morbidity related to pneumonia.

Furthermore, several nutrients are known to be associated with the development of pneumonia. Among other factors, the swallowing reflex is known to be depressed in patients with hypofolatemia below 3 ng/mL, as folic acid deficiency causes impaired metabolism of dopamine in the central nervous system. Supplementation with folic acid, accompanied by normalization of the plasma concentrations of homocysteine and folate, may improve the elicitation of the swallowing reflex [67].

Oral, tube (nasal tube feeding, gastrostomy), central venous, and peripheral venous nutrition nutritional options may be considered for patients who cannot receive adequate nutrition due to dysphagia. In a comparison of nutritional modalities, including oral, tube, and central venous nutrition options in bed-ridden elderly patients with dysphagia, tube feeding led to the longest survival (median, 23 months) [68]. Nutritional support by tube feeding reduces pneumonia-related morbidity and mortality, although it does not eliminate all cases of pneumonia, as compared with other means of nutrition or no nutritional support itself [69].

#### 4.2.5. Swallowing Rehabilitation

While swallowing exercises such as the Mendelsohn manoeuvre, tongue-hold swallowing, supraglottal swallowing, shaker exercises, and effort pitch glide, as well as expiratory muscle strength training, have been shown to improve the structural excursion of each component, there have been no reports of reduced incidence of pneumonia with each exercise [70]. However, in a randomized control study of dysphagia patients with acute stroke, an intensive swallowing rehabilitation program using swallowing exercise therapy combined with a texture-modified diet showed a reduced incidence of under-nutrition and pneumonia [71]. Comprehensively, swallowing therapy including transcranial magnetic stimulation, transcranial direct current stimulation neuromuscular, electrical stimulation, and pharyngeal electrical stimulation has been reported to be effective in reducing the incidence of chest infection or pneumonia in the elderly with oropharyngeal dysphagia [72].

#### 4.2.6. Oral Care

It is now common knowledge that oral care is effective in preventing the onset of pneumonia. In particular, an initial ground-breaking study, executed 20 years ago, showed that an oral care intervention for 2 years in institutionalized elderly people reduced the incidence of pneumonia in both dentulous and edentulous institutional elderly people [73]. Additionally, the cognitive function remained unchanged in the oral care intervention group compared to the non-intervention group. Further, in another one-month randomized controlled trial study conducted in a geriatric ward, the swallowing reflex latency shortened to a plateau after 10 days of oral care intervention, and the cough reflex sensitivity improved to the normal range after approximately one month [74,75]. In other words, oral care is not only about hygiene, but brushing itself can be considered a mechanical stimulus to gingival sensory nerve endings, and consequently to the central nervous system. Research reports have shown that nociception projects to the primary somatosensory cortex, as well as the cingulate and insular cortex (medial pain system), potentially supporting these results [76]. Furthermore, a one-year prospective cohort study of 343 nursing home residents has shown that residents with *Prevotella*, *Veillonella*, and *Treponema* spp. on their tongue coating, using a terminal restriction fragment length polymorphism (TRFLP) analysis, were pre-disposed to developing pneumonia [77]. Additionally, moisture retention on the tongue surfaces of those who developed pneumonia with these detected oral bacteria was low [77]. In other words, lower moisture retention may facilitate the growth of these bacteria associated with the development of pneumonia. Therefore, keeping the tongue moist with moisturising gel, together with oral brushing, may be expected to reduce the days of pyrexia and antimicrobial use, as well as the number of cases of pneumonia [78].

#### 4.2.7. Sitting and Holding Position after Meals

AsP is most common in bed-ridden elderly patients. Many bed-ridden patients are unable to turn over on their own and lie in a supine position. Aspiration of the stomach contents, which can cause pneumonia, is less likely to occur in the semi-recumbent position than in the supine position [79,80]. Positions with a head angle of 30 degrees or more also reduce hospital-acquired pneumonia [81]. In bed-ridden institutionalized elderly patients, a 2 h post-prandial intervention in which the patients were placed at an angle of 30 degrees or more after a meal significantly reduced the number of fever days compared to the group without post-prandial intervention. Therefore, elderly people should be placed in a semi-recumbent position at 30° or higher for 2 h after eating [82]. A comparative study of positional changes showed that the semi-recumbent position reduced the frequency and risk of nosocomial pneumonia, especially in patients on enteral feeding [83].

Subsequently, the prone position has been advocated to prevent gastric reflux, inflammatory reactions, and alveolar over-expansion, as well as providing ventilation efficiency and oxygenation [84]. Therefore, in bed-ridden patients, the prone position may be more protective against the development of pneumonia than supine and even semi-recumbent positions.

#### 4.2.8. Bowel Movement Control

Intestinal dysmotility is common in the elderly. Fecal impaction or stubborn constipation often leads to vomiting of gastrointestinal tract contents, resulting in the development of chemical pneumonitis. A high fiber content and moderate exercise are important for adequate control of bowel movements.

The gut–lung axis is not only physiological, but also involves microbial cross-talk [85]. Gut microbes in pulmonary host defence show that tonic signaling of pattern recognition receptors by microbial products in the steady state contributes to chronic lower respiratory diseases. Gut microbes act in the host’s lung defence, and it has been reported that the tonic signal transduction when microbial products act on pattern recognition receptors contributes to chronic lower respiratory disease. High-fiber diets possibly increase the ratio of *Bacteroidetes* to *Firmicutes* species, and in consequence the production of short chain-fatty acids, which weaken the inflammation by activating GPR-40. It has been established that a high-fiber diet and short chain-fatty acids improve the intestinal microbial composition.

In clinical settings, most patients with recurrent AsP have difficulty eating sufficient fiber due to dysphagia, as well as having difficulty performing adequate exercise due to physical disability. Based on the above, the use of laxatives (as appropriate) may also be effective. Intervening with laxative medication in bed-ridden elderly patients without defecation for several days has been shown to reduce the incidence of pneumonitis but not pneumonia [28].

### 4.3. Strategies for Preventing Aspiration Pneumonia

Deterioration of the swallowing and coughing reflexes and low nutrition have been shown to be predictors of death within 90 days of AsP [46]. A disease time-axis for AsP and dysphagia has recently been identified (Figure 2). Therefore, to reduce the morbidity and mortality associated with AsP, priority should be given to improving the sensitivity of the swallowing reflex and cough reflex, as well as the nutritional status.

**Figure 3:**
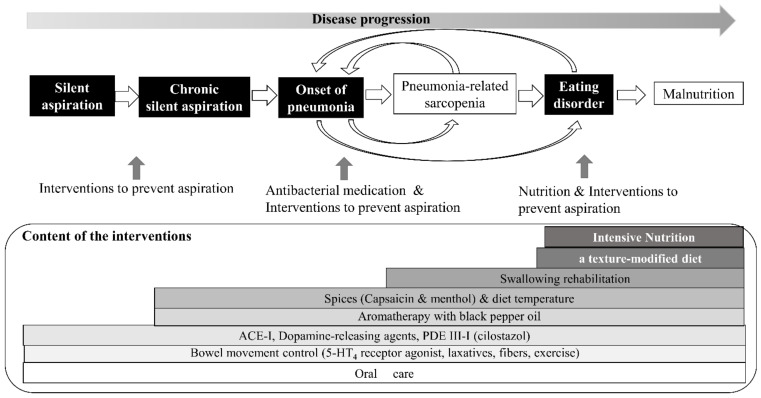
Disease time-axis and comprehensive approaches.

To begin, oral medicines are often difficult to administer to patients with dysphagia. Therefore, the following steps of resuming feeding and eating are recommended for patients at high risk of AsP. As a first step, during the acute fasting phase of antimicrobial treatment, aromatherapy intervention should be applied to activate the insular cortex, followed by a pureed or nectar-thickened meal containing capsaicin or menthol, in order to provoke both the swallowing reflex and cough reflex sensitivity levels. ACE-I, a dopamine-releasing agents, and phosphodiesterase-III inhibitors can be administered while gradually increasing the physicality of the meal. Oral care and swallowing rehabilitation should be consistently performed from the time of fasting. In our self-controlled case series, the intervention led to a significant decrease in the onset of pneumonia after the resumption of eating, which may indicate a certain effect (Figure 3) [86].

Additionally, when a patient is unable to intake sufficient calories and nutrients by mouth, it is recommended to provide essential nutrition through various nutritional routes, such as oral, gastrostomy, transcentral venous, or peripheral transvenous routes, with the step-wise eating and feeding methods mentioned above. This approach has the potential to reduce recurrent AsP and slow down the progression of dysphagia, thereby postponing the time to death. In clinical settings, we assume that while the state with inability to intake adequate nutrition orally stands as a gateway to the end of life for the elderly, there are many elderly individuals who may be able to escape from this gateway through nutritional support. Viewed another way, patients who have difficulties receiving essential nutrition due to diarrhoea during gastrostomy feeding, liver dysfunction, and repeated catheter-related bloodstream infections during central-venous nutrition may truly be at the end of their life.

## 5. Conclusions

There exists a pathophysiological time-axis of disease involving aspiration only, AsP, pneumonia-related sarcopenia, and eating disorders. Therefore, it is important to diagnose the position of an individuals on the disease time-axis in order to provide appropriate individualized treatment and prevention, taking into account their wishes and quality of life. Preventive measures to improve the upper airway reflex should be implemented from the disease onset to the end of life. Finally, education regarding how to prevent aspiration at home is important for both patients and their families and carers (see Table 1).

## Figures and Tables

**Table 1:** How to prevent aspiration at home.

*Before Eating*
Swallowing rehabilitation Aromatherapy (i.e., with black pepper oil)
***Meals***
Spices (capsaicin and/or menthol) Diet temperature Texture-modified diet Fiber
***After Eating***
Oral care A semi-recumbent position at 30° or higher for 2 h Laxatives (if appropriate)

## Data Availability

The report did not report any data.
